# Parental satisfaction with quality of neonatal care in different level hospitals: evidence from Vietnam

**DOI:** 10.1186/s12913-020-5070-5

**Published:** 2020-03-20

**Authors:** An Thi Binh Nguyen, Ngan Thi Kim Nguyen, Phuc Huu Phan, Peter van Eeuwijk, Günther Fink

**Affiliations:** 1 Swiss Tropical and Public Health Institute, Basel, Switzerland; 2 University of Basel, Basel, Switzerland; 3 Hanoi University of Public Health, Hanoi, Vietnam; 4 Vietnam National Children’s Hospital, Hanoi, Vietnam; 5 Institute of Social Anthropology, Basel, Switzerland

**Keywords:** Vietnam, Preterm infants, Parental satisfaction, Health facility ranking

## Abstract

**Background:**

Most health systems provide the most specialized, and presumably also the highest quality of care at a central level. This study assessed parental satisfaction and its determinants in the context of neonatal care in a provincial as well as a national hospital of Vietnam.

**Methods:**

In this cross-sectional quantitative study, parents of 340 preterm infants admitted to neonatal care units of a national and a provincial hospital in 2018 were interviewed using structured questionnaires. Unadjusted and adjusted linear regression models were used to assess the relationship between parental satisfaction and hospital rank.

**Results:**

The mean parental satisfaction score was 3.74 at the provincial, and 3.56 at the national hospital. These satisfaction differences persisted when parent and child characteristics were adjusted for in multivariate analysis. Longer length of stay and worsening infant health status were associated with parents reporting lower levels of satisfaction with the quality of care being provided at the healthcare facility.

**Conclusions:**

This study suggests that parents of preterm infants admitted in a provincial hospital were more satisfied with the quality of care received than those in a specialized national hospital. Length of stay and infant health status were the two most important determinants of level of parental satisfaction.

## Background

Perceived quality of health care services plays an important role in the development and improvement of health services. Patients who are satisfied with the quality of care received have been shown to comply more with medical treatment protocols and also to recommend clinical care to others [1]. Achieving full patient satisfaction requires fulfilling expectations, needs, and/or desires with regard to health care [2].

Given that infants cannot express their particular health needs and demands, experiences, opinions and satisfaction of parents play a vital role in choosing the most appropriate healthcare for children [3, 4]. Studies on parent’s satisfaction with paediatrics care have highlighted numerous domains as critical for caregiver satisfaction, including interpersonal relationships, accessibility of services, and provision of information on health and decision-making processes. For Neonatal Care Unit (NCU) services, communication and information sharing, emotional support, family involvement, treatment skill and environmental conditions have been shown to be particularly important [3, 5–9]. Socio-economic factors that affect parental satisfaction are diverse from different studies. In the literature, age, sex, level of education, and income of parents have been documented to be determinant factors [8–12].

In Vietnam, most past patient satisfaction research has focused only on adult patients in general hospitals. Results from studies in general hospitals suggest waiting time, attitude of health workers, and communication of health staff with patients as key factors for patient satisfaction [13–15]. In accordance with the Vietnamese health insurance law (2014), all children under 6 years of age are automatically covered by the national health insurance. Preterm infants as well as infant with special health care needs are generally either treated at provincial (regional) or national hospitals. To the best of our knowledge, there are no studies examining parent satisfaction with neonatal care in these two settings in Vietnam. In this study, we aim to assess and compare parental satisfaction and its determinants in a two purposely chosen facilities: the provincial hospital of Thanh Hoa province, and the national hospital in Hanoi Capital.

## Method

### Study design

This is a cross-sectional, quantitative study using a structured questionnaire to interview parents of premature infants in the Neonatal Care Units of a provincial and a more specialized (higher level) national hospital. The data collection period lasted from January to June 2018.

### Study setting

The national hospital system of Vietnam is divided into four levels: a national, a provincial, a district and a community level based on the difference in number of hospital beds, health workers, medical technologies and medical services provided. Therefore, at the community level, only very basic services provided while the national level hospitals offer the most specialized care and are designed to provide the highest quality of care in the country.

### Study sites

Since most preterm infants with specialized care needs are treated either at provincial or at national hospitals, we purposively selected one large hospital from each level for this study: Vietnam National Children’s Hospital (VNCH) and Thanh Hoa Pediatric Hospital (THPH).

Thanh Hoa Pediatric Hospital is the only provincial public pediatric hospital in Thanh Hoa province with 1050 beds. Thanh Hoa province is located in the North Central Coast region of *Vietnam* and comprises a total population of 3.5 million people across 24 districts. The primary economic activities in this province are agriculture, forestry, fishery, and tourism. It is also an industrial center [16]. The neonatal care unit of the hospital admits an average of 200 infants and 22 preterm infants per month. The infant-nurse and infant-doctor ratios in the neonatal care unit were 4.6 and 18.2 in 2018, respectively.

Vietnam National Children’s Hospital is a national referral hospital located in Hanoi City, the capital of Vietnam. The hospital is the largest pediatric hospital in Northern Vietnam with 1900 beds. It is the primary referral hospital for all 38 provinces of northern Vietnam (with a total population of 43 million people) as well as a center for research, teaching, and postgraduate training in newborn diseases. The neonatal care unit of this hospital is responsible for the diagnosis, treatment, and care of all premature infants and newborns referred to from lower-level facilities, including all provincial hospitals for specialized care. The estimated average number of neonates and preterm infants admitted in this unit are around 450 neonates and 110 infants per month. The infant-nurse and infant-doctor ratios in the neonatal care unit were 4.8 and 22.5 in 2018, respectively.

### Participants

We interviewed parents (mother or father) of preterm babies admitted to the two neonatal care units for 6 months, from January to June of 2018. The inclusion criteria for interviewees were that parents were at least 18 years old and literate, that their child was being treated and had stayed at least 7 days in the neonatal care unit at the time of interview, and they were willing to participate in the study.

### Sample size and sampling methods

Based on the list of preterm infants admitted in neonatal care unit each week, we met parents in neonatal care units of two hospitals and invited them to participate in our study. If they were willing to participate in the study, parents selected a convenient place for the interview, often outside the neonatal care unit, and filled out the study questionnaire. If both parents were available, the mother was given preference and only she was interviewed. The interview was conducted by members of our research team.

We interviewed all parents who have met the above criteria between January and June 2018. After 6 months of data collection, the total number of parents participated in our study was 340 parents: 90 parents in Thanh Hoa Pediatric Hospital and 250 parents in Vietnam National Children’s Hospital.

### Data collection tools and variables

#### Data collection tools

Data was collected through a two-part questionnaire: the first part comprised 13 questions capturing socio-economic characteristics of parents and clinical characteristics of preterm infants. The second part of the questionnaire comprised of 22 satisfaction questions covering three dimensions: care and treatment, communication, and hospital environment measuring parental satisfaction on a five-point Likert scale. Level of agreement for each statement in the questionnaire was scored from 1 to 5 with 1 indicating the lowest level of agreement (totally disagree) and 5 indicating the highest level of agreement (totally agree). The questionnaire was developed for this study (see Additional file 1) and the first version of the questionnaire was based on McPherson’s Parental Satisfaction Survey (PSS) [17] and EMPATHIC questionnaire [18], which both measure parent satisfaction in paediatric intensive care units. Back-forward translation was conducted and then we dropped questions inappropriate for the context of Vietnamese hospitals. This second version was pilot-tested with 30 parents in both hospitals to make sure that respondents understand the questions clearly and the questions covered all satisfaction areas. There were no changes in the contents of the second version, only few words had been changed after the pilot test.

Cronbach’s alpha for the adapted questionnaire was 0.93: For the 7 items in the care and treatment component, Cronbach’s alpha was 0.84; for the 12 items on the communication scale and the 3 items on the hospital environment scales Cronbach’s alpha was 0.90 and 0.76, respectively.

##### Outcome variable

The primary outcome of interest was parental satisfaction with the level of care provided. Parental satisfaction was modeled as the average satisfaction score measured across the 22 questions asked.

##### Covariate variables

Social-demographic characteristics of parents as well as clinical characteristics of premature babies included: gender of respondents (female, male); marriage status (married, divorced/separated); parental age group (15–25, 26–35, 35+ years); ethnicity (Kinh, others); level of education (high school and lower, undergraduate and postgraduate); job (farmer/fisherman/worker, officer, freelancer, students); residence (rural, urban); income per month (≤1.950.000 VND, > 1.950.000 VND); gender of the child (female, male); gestational age (32–37, 28–31, < 28 weeks); birth weight (≥2500 g, 1500-2499 g, 1000-1499 g, < 1000 g); health status of the infant (improved, no change or worsen); and length of stay (≤14 days, > 14 days).

### Data analysis

Firstly, descriptive statistics (percentage, mean, min, max) for all variables of interest were calculated. Following this, we estimated the average difference in satisfaction score between caregivers at the provincial and caregivers at the national hospital, both unconditional and conditional on all infants’ and parents’ characteristics. The main independent variable of interest for this analysis was a dichotomous variable for treatment at the national hospital, with the provincial hospital serving as reference group. Thirdly, we estimated the same differences by population group. Our data was stratified according to income, length of stay, and health status of infants. We then estimated the difference in satisfaction between two hospitals adjusting for parent and child characteristics.

## Results

After 6 months of data collection, data from 340 parents were included in the study, 90 of which had infants admitted to Thanh Hoa Pediatric Hospital (26.5%) and 250 parents with infants admitted to Vietnam National Children’s Hospital (73.5%). Table 1 provides characteristics of the parents and the infants. Most of the participants were female (61.2%) and married (98.5%). Parents aged between 26 to 35 years old constituted 60% of the respondents; 25.6% of parents were aged between 15 and 25 years, and 14.4% of parents were 35 years of age and older. More than half of the parents (52.1%) had high school or lower level education. A greater percentage of respondents (66.8%) reported an income over 1.950.000 VND (USD 85/per month); and 60.6% of the infants were male. More than half of the infants (58.2%) had a birth weight between 1500 and 2499 g; and 3% were under 1000 g. Two-thirds of the infants (68.2%) were admitted to hospitals for less than 14 days and in 61.5% of the cases, parents reported that the condition of their infants had improved since admission.

**Table 1:** Characteristics of participants (parents and infants)

Characteristics	Hospital (n, %)		Total	
***Socio-economic characteristics of parents***	Thanh Hoa Pediatric Hospital (***n*** = 90)	Vietnam National Children’s Hospital (***n*** = 250)	Frequency (***N*** = 340)	Percentage (%)
**Gender of respondent**				
Male	48 (53.3)	84 (33.6)	132	38.8
Female	42 (46.7)	166 (66.4)	208	61.2
**Marriage status**				
Married	88 (97.8)	247 (98.8)	335	98.5
Divorced/Separated	2 (2.2)	3 (1.2)	5	1.5
**Age**				
15–25	12 (13.3)	75 (30)	87	25.6
26–35	62 (68.9)	142 (56.8)	204	60.0
35+	16 (17.8)	33 (13.2)	49	14.4
**Ethnicity**				
Kinh	77 (85.6)	200 (80)	277	81.5
Others	13 (14.4)	50 (20)	63	18.5
**Level of education**				
High school and lower	32 (35.6)	145 (58.0)	177	52.1
Undergraduate and higher	58 (64.4)	105 (42.0)	163	47.9
**Job**				
Farmer/fisherman/worker	33 (36.7)	106 (42.4)	139	40.9
Officer	32 (35.6)	57 (22.8)	89	26.2
Freelancer/self-employed	20 (22.2)	77 (30.8)	97	28.5
Student	5 (5.6)	10 (4.0)	15	4.4
**Residence**				
Rural	40 (44.4)	128 (51.2)	168	49.4
Urban	50 (55.6)	122 (48.8)	172	50.6
**Income (per month)**				
> 1.950.000 VND (>USD 85)	77 (85.6)	150 (60.0)	227	66.8
≤ 1.950.000 VND (≤USD 85)	13 (14.4)	100 (40.0)	113	33.2
***Characteristics of preterm infants***				
**Gender**				
Male	56 (62.2)	150 (60.0)	206	60.6
Female	34 (37.8)	100 (40.0)	134	39.4
**Gestational age**				
32–37 weeks	52 (57.8)	94 (37.6)	146	42.9
28–31 weeks	30 (33.3)	120 (48.0)	150	44.1
< 28 weeks	8 (8.9)	36 (14.4)	44	13.0
**Birth weight**				
≥ 2500 g	13 (14.4)	22 (8.8)	35	10.3
1500-2499 g	67 (74.5)	131 (52.4)	198	58.2
1000-1499 g	8 (8.9)	89 (35.6)	97	28.5
< 1000 g	2 (2.2)	8 (3.2)	10	3.0
**Health status of the infant (at the interview time relative to admission)**				
Improved	68 (75.6)	141 (56.4)	209	61.5
No change or worsened	22 (24.4)	109 (43.6)	131	38.5
**Length of stay of the infant (LOS) (at the interview time)**				
**<** 14 days	79 (87.8)	153 (61.2)	232	68.2
≥ 14 days	11 (12.22)	97 (38.80)	108	31.76

Table 2 describes mean parental satisfaction by the hospital. Average satisfaction scores were 3.74 and 3.56 at the regional and national hospitals, respectively. In both hospitals, parents were most satisfied with the information from doctors regarding the expected health outcomes of their infants (3.99 over 5). In this domain, the provincial hospital scored lower (3.92) than the national hospital (4.02). Participants were less satisfied with the manner in which doctors and nurses were introduced to them (2.87 overall) and information related to who was responsible for their child (3.00 overall). For most other aspects of health services, satisfaction scores at the regional hospital were higher than the scores at the national hospital.

**Table 2:** The satisfaction of parents with health services

***Content (n = 340)***	Thanh Hoa Pediatric Hospital (***n*** = 90)		Vietnam National Children’s Hospital (***n*** = 250)		***Total (N = 340)***	
	***Mean (Min-Max)***	***SD***	***Mean (Min-Max)***	***SD***	***Mean (Min-Max)***	***SD***
**Care and treatment**						
At admission our child’s medical history was known by the doctors and nurses.	4.01 (3–5)	0.32	3.74 (1–5)	0.74	3.81 (1–5)	0.66
During acute situations there is always a nurse to support us promptly.	4.02 (3–5)	0.33	3.78 (1–5)	0.75	3.84 (1–5)	0.67
The correct medication is always given on time.	4.02 (3–5)	0.30	3.65 (1–5)	0.75	3.75 (1–5)	0.68
Our child is always well taken care of by the nurses while in the incubator/bed.	3.98 (3–5)	0.37	3.45 (1–5)	0.80	3.58 (1–5)	0.75
The health staff team cares about my child’s needs and about us.	3.79 (3–5)	0.49	3.34 (1–5)	0.86	3.46 (1–5)	0.80
Every day we know who of the doctors and nurses was responsible for our child.	3.30 (1–5)	0.79	2.90 (1–5)	1.04	3.00 (1–5)	0.99
I would recommend this NCU to friend or family member who needed to be hospitalized.	3.68 (3–5)	0.56	3.76 (1–5)	0.72	3.74 (1–5)	0.68
**Communication**						
We are given clear information about our child’s disease.	3.91 (1–5)	0.47	3.82 (1–5)	0.77	3.84 (1–5)	0.71
The doctor clearly informs us about the consequences of our child’s treatment.	3.93 (3–5)	0.42	3.78 (1–5)	0.79	3.82 (1–5)	0.72
We receive clear information about the examinations and tests.	3.49 (2–5)	0.64	3.48 (1–5)	0.87	3.48 (1–5)	0.81
We receive understandable information about the effects of the drugs.	3.67 (2–5)	0.56	3.46 (1–5)	0.78	3.52 (1–5)	0.73
The doctor informs us about the expected health outcomes of our child.	3.67 (3–5)	0.52	3.70 (1–5)	0.74	3.69 (1–5)	0.69
We are always informed right away when our child’s physical condition worsened.	3.92 (3–5)	0.48	4.02 (1–5)	0.67	3.99 (1–5)	0.62
My child’s privacy and confidentiality are respected during his/her NCU stay.	4.03 (3–5)	0.32	3.95 (1–5)	0.70	3.97 (1–5)	0.62
The information provided by the doctors and nurses is understandable.	3.91 (3–5)	0.36	3.61 (1–5)	0.71	3.69 (1–5)	0.65
Our questions are clearly answered.	3.61 (3–5)	0.53	3.4 (1–5)	0.84	3.45 (1–5)	0.77
The doctors and nurses always take time to listen to us.	3.41 (2–5)	0.62	3.19 (1–5)	0.89	3.24 (1–5)	0.83
We receive sympathy from the doctors and nurses.	3.37 (2–5)	0.57	3.23 (1–5)	0.92	3.27 (1–5)	0.84
Nurses and doctors always introduce themselves by name and function.	2.69 (1–5)	0.81	2.94 (1–5)	0.96	2.87 (1–5)	0.93
**Hospital environment**						
My child’s room/incubator is clean and comfortable.	3.93 (3–5)	0.32	3.78 (1–5)	0.62	3.82 (1–5)	0.56
There is enough space around our child’s incubator/bed.	4.01 (3–5)	0.44	3.75 (1–5)	0.68	3.82 (1–5)	0.64
My child’s room is quiet enough for him/her to rest.	3.90 (2–5)	0.58	3.70 (1–5)	0.97	3.75 (1–5)	0.89
**Overall satisfaction score**	**3.74 (3–5)**	0.25	**3.56 (1–4.95)**	0.53	3.61 (1–5)	0.48

Table 3 shows the main regression results. Compared to the provincial hospital, average satisfaction scores were 0.18 points lower at the national hospital (95% CI: − 0.29, − 0.06, *P* < 0.05). After controlling for the child and parents’ covariates displayed in Table 1, the estimated difference declined marginally to − 0.13 (95% CI: − 0.26, − 0.01; *p* < 0.005).

**Table 3:** Hospital level predicting parental satisfaction

Characteristics	Overall satisfaction score			
	Unadjusted regression coefficient (95% CI) (1)	***P***	Adjusted regression coefficient (95% CI) (2)	***P***
**Hospital (main covariate)**				
Thanh Hoa Pediatric Hospital	Ref	–	Ref	–
Vietnam National Children’s Hospital	**−0.18 (− 0.29, − 0.06)**	**0.003**	**− 0.13 (− 0.26, − 0.01)**	**0.03**
**Parent’s gender**				
Male			Ref	–
Female			0.05 (−0.06, 0.15)	0.38
**Education (main covariate)**				
High school and lower			Ref	–
Undergraduate and higher			0.06 (−0.07, 0.19)	0.35
**Marriage status**				
Married			Ref	–
Divorced/Separated			0.02 (−0.38, 0.42)	0.91
**Age**				
15–25			Ref	–
26–35			0.03 (−0.09, 0.16)	0.59
35+			−0.04 (− 0.21, 0.13)	0.62
**Ethnicity**				
Kinh			Ref	–
Others			0.03 (−0.12, 0.18)	0.69
**Job**				
Farmer/fisherman/worker			Ref	–
Officer			0.15 (−0.009, 0.31)	0.07
Freelancer			**0.16 (0.04, 0.27)**	**0.01**
Students			0.03 (−0.25, 0.30)	0.84
**Residence**				
Urban			Ref	–
Rural			0.06 (−0.06, 0.18)	0.33
**Income (per month)**				
≤ 1.950.000 VND (>USD 85)			Ref	–
> 1.950.000 VND (≤USD 85)			0.06 (− 0.07, 0.18)	0.40
**Infant’s gender**				
Female			Ref	–
Male			0.07 (−0.03, 0.16)	0.18
**Gestational age**				
< 28 weeks			Ref	–
28–31 weeks			0.06 (−0.10, 0.22)	0.46
32–37 weeks			0.06 (−0.12, 0.24)	0.52
**Birth weight**				
≥ 2500 g			Ref	–
1500-2499 g			−0.01 (−0.19, 0.16)	0.88
1000-1499 g			0.04 (−0.17, 0.25)	0.69
< 1000 g			0.09 (−0.26, 0.44)	0.61
**Health status of the infant (at the interview time)**				
No change or worsened			Ref	–
Improved			**0.32 (0.22, 0.43)**	**< 0.001**
**Length of stay of the infant (at the interview time)**				
**<** 14 days			Ref	–
14–29 days			**0.12 (0.008, 0.23)**	**0.04**

*Ref* Reference group

Table 4 depicts the stratified regression results. When we stratified parents by income group, we found larger differences in satisfaction among lower income parents, even though the differences in estimated effect sizes are not statistically significant (Table 4, columns 1 and 2). When we stratify our analysis by duration of stay at the hospital, we found greater gaps for children with shorter stays (Table 4, columns 3 and 4). Lastly, when we stratified by health trajectory, we found larger gaps in satisfaction scores for parents of children not recovering (columns 5 and 6). Once again, confidence intervals overlapped for the subgroup estimates. Across all the subgroups analyzed, the national hospital received lower satisfaction scores than the regional one in the fully adjusted models.

**Table 4:** Stratified association between parental satisfaction score and participant characteristics (parent and infant)

Characteristic	Income		Length of stay		Health status	
	Under average income (1)	Above average income (2)	< 14 days (3)	≥14 days (4)	Improved (5)	No change or worsen (6)
**Hospital (main covariate)**						
Thanh Hoa Paediatric Hospital	Ref	Ref	Ref	Ref	Ref	Ref
Vietnam National Children’s Hospital	−0.28 (− 0.56, 0.005)	−0.08 (− 0.22, 0.06)	−0.15 (− 0.27, − 0.02)*	−0.07 (− 0.42, 0.28)	−0.03 (− 0.17, 0.12)	−0.36 (− 0.59, 0.13)*

Covariates in models: Parent’s gender, education, marriage status, age, ethnicity, job, place of residence, income (model 3, 4, 5, 6), infant’s gender, gestational age, birth weight, health status (model 1, 2, 3, 4), LOS (for model 1, 2, 5, 6)

*Ref* Reference group

*: P < 0.05

## Discussion

Parental choices are particularly important for preterm infants who depend on receiving the highest quality care for their survival and wellbeing. Considering this, the main results of this study are troubling as they suggest that, on average, parents are more satisfied with the provincial than with the national hospital. This disparity may be partially explained by the fact that the national hospital is the primary referral hospital for 38 provinces, resulting in a heavy workload, more impersonal communication, and limited time for counseling the parents. Indeed, findings from previous studies show that satisfaction is strongly affected by institution-related characteristics such as the patient-nurse ratio of the hospital [19–22], hospital located in different regions [5], hospital reputation [23], hospital size [22, 24], and type of hospital ownership [25].

Even though the infant-nurse ratio (4.6 in Thanh Hoa Pediatrics Hospital and 4.8 in Vietnam National Children’s Hospital) and the infant-doctor ratio (18.2 in Thanh Hoa Pediatrics Hospital and 22.5 in Vietnam National Children’s Hospital) looked similar in the two hospitals, we found that parents rated the communication skills of health professionals in the national hospital lower than those of provincial hospital staff. However, the national hospital treats more severe cases with longer length of stays than the provincial hospital; thus, their doctors and nurses are likely to be exposed to high-pressure work condition and long working hours. Direct, personal and proactive communication of health staff with parents can reduce stress and anxiety for parents [3, 6, 7, 25], but may of course be hard to be performed for staff in a crowded national hospital.

Our data collected as part of this study shows that infants in the national hospital suffer, on average, from more severe conditions and, therefore, often have to be admitted to this hospital for longer periods. On average, children at the national hospital are also more likely to be born prematurely, and to show low birth weight. This additional medical care required for these children may at least partially explain the lacking satisfaction of parents at national hospitals as suggested in the related literature [3, 5, 8, 9, 22, 26]. On average, caregivers at the national hospital were also less educated, which may result in different understanding of, or responses to questions. However, differences in parental satisfaction remained statistically significant even when both health and socioeconomic variables were controlled for in the adjusted models.

Furthermore, our study suggests that even though the difference in satisfaction between two hospitals was not statistically significant in both income groups, there was a larger satisfaction difference among parents in the lower income group. This finding contradicts what has been found in other studies, which show a significant association between income and satisfaction, with lower income parents reporting lower levels of satisfaction [9]. This may be explained by the fact that in Vietnam care provision for premature infants is fully covered by the national health insurance. Thus, all infants are given the best possible treatment irrespective of the parents’ level of income which leads finally to an overall high rate of satisfaction among parents from lower income groups.

This study had several limitations. Firstly, the study was conducted in only two hospitals. While these hospitals were purposely sampled to represent the national and provincial level, they may not be representative of all hospitals of a similar level in the country. Secondly, the study was conducted when the infants were currently being treated in hospitals. Therefore, parents may hesitate to provide critical assessments of the hospitals’ health services, increasing the risk of a reporting bias.

## Conclusions

The results presented in this study suggest that the parental satisfaction with quality of neonatal care is higher at the provincial level hospital than at the national level hospital observed. These differences appear to partially reflect differences in parental background as well as severity of health problems, but persisted even when parent and child characteristics were adjusted for in multivariate analysis.

Improving provider-parent interactions, in particular through more personalized communication and counseling could help improving parental satisfaction with pediatric care at national hospitals in Vietnam.

## Supplementary information

**Additional file 1.** The English language version of the questionnaire for this study

## Data Availability

The datasets analyzed for this study are not publicly available due to given approval limited to their use only for this study but are available from the corresponding author on reasonable request.

## Abbreviations

NCU: Neonatal Care Unit
VNCH: Vietnam National Children’s Hospital
THPH: Thanh Hoa Pediatric Hospital
