# Supplementary material for: Parental satisfaction with quality of neonatal care in different level hospitals: evidence from Vietnam
Source: BMC Health Serv Res. 2020 Mar 20;20:238. doi: 10.1186/s12913-020-5070-5 (PMC7082960; doi:10.1186/s12913-020-5070-5)
Supplement: Supplementary file 1 — Additional file 1. The English language version of the questionnaire for this study [file 12913_2020_5070_MOESM1_ESM.docx]

**Additional file 1**

**Structured questionnaire**: **Parental satisfaction with quality of neonatal care in different level hospitals: Evidence from Vietnam**

Code of form:

Date of interview:

Hospital:

*Guidance:*

*Circle on the appropriate number or write in the blank line*

*Use a pen, not pencil and avoid erasing*

1. **Characteristic of parents and preterm infants**
2. **Socio-economic and demographic characteristic of parents**

| **Number** | **Content** |  | **Note** |
| --- | --- | --- | --- |
| 1.1 | Gender  *Male*  *Female* | 0  1 |  |
|  | Marriage status  *Married*  *Divorced/ Separated* | 0  1 |  |
| 1.2 | Age | ………… | *Sun calendar* |
| 1.3 | Ethnicity  *Kinh*  *Other ethnic group* | 1  ….. | *Please indicate* |
| 1.4 | Education  *Primary*  *Secondary school*  *High school*  *Undergraduate*  *Post- graduate*  *Other* | 1  2  3  4  5  99 |  |
| 1.4 | Job  *Famer/fisherman/worker*  *Officer*  *Freelancer*  *Student*  *Others (please indicate)* | 1  2  3  4  5 |  |
| 1.5 | Residence  *Rural*  *Urban* | 1  2 |  |
| 1.6 | Income(per month)  *<1 million (Vietnam Dong)*  *1-1.3 million*  *1.3-1.95 million*  *>1.95 million* | 1  2  3  4 |  |

1. **Characteristic of preterm infants**

| **Number** | **Content** |  | **Note** |
| --- | --- | --- | --- |
| 2.1 | Gender  *Female*  *Male* | 1  0 |  |
|  | Birth weight  *≥2500 g*  *2499-1500 g*  *1499-1000 g*  *<1000g* | 1  2  3  4 |  |
| 2.3 | Gestational weeks | … |  |
| 2.4 | Length of stay in hospital (days) | …. | At the time of interview |
| 2.5 | Health status of the child after admission in hospital  *Better*  *Unchanged*  *Worsen*  *Other (please indicate)* | 1  2  3  … | Health status at the moment of interview |

**II. The satisfaction of parents**

Totally disagree :1

Disagree: 2

Neutral: 3

Agree : 4

Totally agree : 5

***Guidance: Please circle the most appropriate number of each statement in which correspond most closely to your desired response.***

| **Number** | **Content** | **Level of agreement** | | | | |
| --- | --- | --- | --- | --- | --- | --- |
|  |  | **Totally disagree** | **Disagree** | **Neutral** | **Agree** | **Totally agree** |
| **A** | **Care and treatment** | 1 | 2 | 3 | 4 | 5 |
| 1 | At admission our child’s medical history was known by the doctors and nurses | 1 | 2 | 3 | 4 | 5 |
| 2 | During acute situations there is always a nurse to support us  promptly | 1 | 2 | 3 | 4 | 5 |
| 3 | The correct medication is always given on time | 1 | 2 | 3 | 4 | 5 |
| 4 | Our child is always well taken care of by the nurses while in the incubator/bed | 1 | 2 | 3 | 4 | 5 |
| 5 | The health staff team cares about my child’s needs and about us | 1 | 2 | 3 | 4 | 5 |
| 6 | Every day we know who of the doctors and nurses was responsible for our child | 1 | 2 | 3 | 4 | 5 |
| 7 | I would recommend this NCU to friend or family member who needed to be hospitalized | 1 | 2 | 3 | 4 | 5 |
| **B** | **Communication** |  |  |  |  |  |
| 8 | We are given clear information about our child’s disease | 1 | 2 | 3 | 4 | 5 |
| 9 | The doctor clearly informs us about the consequences of our child’s treatment | 1 | 2 | 3 | 4 | 5 |
| 10 | We receive clear information about the examinations and tests | 1 | 2 | 3 | 4 | 5 |
| 11 | We receive understandable information about the effects of the drugs | 1 | 2 | 3 | 4 | 5 |
| 12 | The doctor informs us about the expected health outcomes of our child | 1 | 2 | 3 | 4 | 5 |
| 13 | We are always informed right away when our child’s physical condition worsened | 1 | 2 | 3 | 4 | 5 |
| 14 | My child’s privacy and confidentiality are respected during his/her NCU stay | 1 | 2 | 3 | 4 | 5 |
| 15 | The information provided by the doctors and nurses is understandable | 1 | 2 | 3 | 4 | 5 |
| 16 | Our questions are clearly answered | 1 | 2 | 3 | 4 | 5 |
| 17 | The doctors and nurses always take time to listen to us | 1 | 2 | 3 | 4 | 5 |
| 18 | We receive sympathy from the doctors and nurses | 1 | 2 | 3 | 4 | 5 |
| 19 | Nurses and doctors always introduce themselves by name and function | 1 | 2 | 3 | 4 | 5 |
| **C** | **Hospital Environment** |  |  |  |  |  |
| 20 | There is enough space around our child’s incubator/bed. | 1 | 2 | 3 | 4 | 5 |
| 21 | My child’s room is clean and comfortable | 1 | 2 | 3 | 4 | 5 |
| 22 | My child’s room is quiet enough for him/her to rest | 1 | 2 | 3 | 4 | 5 |
